# Influence of Pleistocene climate fluctuations on the demographic history and distribution of the critically endangered Chinese pangolin (*Manis pentadactyla*)

**DOI:** 10.1186/s40850-022-00153-6

**Published:** 2022-09-01

**Authors:** Shichao Wei, Song Sun, Hongliang Dou, Fuyu An, Haiyang Gao, Ce Guo, Yan Hua

**Affiliations:** Guangdong Provincial Key Laboratory of Silviculture, Protection and Utilization, Guangdong Academy of Forestry, Tianhe District, Guangshan 1st Road 233, Guangzhou, 510520 China

**Keywords:** Pleistocene climate fluctuations, Last glaciation maximum, endangered species, species distribution model, *Manis pentadactyla*, Complete mitochondrial genome

## Abstract

**Background:**

Pleistocene climate fluctuations have strongly modified species genetic diversity and distributions. The Chinese pangolin (*Manis pentadactyla*) has been recognized as a critically endangered animal due to heavy poaching and trafficking. However, the effect of Pleistocene climate fluctuations on the genetic diversity and spatial distribution of the Chinese pangolin remains largely unknown. Here, we combined whole genome sequencing data, analysis of complete mitochondrial genomes, and a large amount of occurrence data from field surveys to infer the ancestral demographic history and predict the past spatial dynamics of the Chinese pangolin in Guangdong Province, China.

**Results:**

Our results indicated that there were two subpopulations, which showed similar trends of population size change in response to past climatic changes. We estimated a peak effective population size (*N*_e_) during the last interglacial (LIG), followed by a marked decrease (~ 0.5 to fivefold change) until the last glacial maximum (LGM) and a rebound to a small peak population size during the Mid-Holocene (MH). The estimated time of the separation event between two subpopulations was approximately 3,000–2,500 years ago (ka). We estimated that the distribution of suitable areas shrank by 14.4% from the LIG to LGM, followed by an expansion of 31.4% from the LGM to MH and has been stable since then. In addition, we identified an elevational shift and suitable area decreased significantly during the LGM, but that the geographic extent of suitable areas in the western region increased from the LIG to present. The eastern region of Guangdong Province had the highest habitat suitability across all the climate scenarios.

**Conclusions:**

Our results suggested that Pleistocene climate fluctuations played an important role in shaping patterns of genetic diversity and spatial distribution, and that human stressors likely contributed to the recent divergence of two Chinese pangolin subpopulations sampled here. We argue that a key protected area should be established in the eastern region of Guangdong Province. As such, this study provides a more thorough understanding of the impacts of Pleistocene climate fluctuations impacts on a mammalian species in southern China and suggests more robust management and conservation plans for this Critically Endangered species of special interest.

**Supplementary Information:**

The online version contains supplementary material available at 10.1186/s40850-022-00153-6.

## Background

Pleistocene climate fluctuations are predicted to have ongoing implications for genetic variation, relative abundance, seasonal timing, and the geographic range of terrestrial organisms [1–4]. In particular, the last glacial maximum (LGM, approximately 21 thousand years ago, ka) represents one of Earth’s most extreme periods of environmental variability, with intensive cooling and drying climate [5]. At that time, the populations of many taxa were suffering from stressful environmental conditions, resulting in sharp decreases in population genetic diversity and areas of suitable habitats [3, 6]. A combination of the genetic data and distribution data carries rich information about a species old and recent evolutionary history, and can help us to develop effective conservation strategies [1, 7, 8].

Southern China is a biodiversity hotspot in low-latitude East Asia, harbouring a high proportion of endemic species and extensive biodiversity [9]. Unlike Europe and North America, most of southern China was not covered by ice during the Pleistocene [10, 11]. However, temperatures were 2–4 °C colder than they are today during the last glacial maximum (LGM) on the East Asian continent [12, 13]. Previous studies showed that Quaternary climatic fluctuations might have had less of an impact on patterns of genetic diversity in southern China than in Europe and North America, and their impact within these regions might have been heterogeneous [14–16]. However, most of these studies have only focused on investigating the effects of climate in driving current patterns of genetic diversity within species in southern China [14–16], but the shifts in species’ distribution ranges have rarely been investigated due to a lack of accurate occurrence data [17].

The Chinese pangolin (*Manis pentadactyla*), distributed in southern China [18], has been recognized as the most heavily poached and trafficked animal worldwide [19] and is classified as critically endangered on the IUCN Red List. Its habitats have been destroyed due to urbanization, infrastructure development, and monoculture forest plantations [18]. Extensive population declines of up to 94% occurred from the 1960s to the early 2000s [20]. Moreover, it is suspected that populations have experienced a 90% decline over the last 20 years, and a further decline of 90% is predicted over the next twenty years [19]. To date, genomic studies of Chinese pangolins from Taiwan and Yunnan Province have revealed long-term population size fluctuations and the recent population decline because of long-term environmental changes and recent human activities, respectively [21, 22]. However, in other provinces within the geographic distribution, how the genetic diversity of their populations responded to the effect of Pleistocene climate fluctuations remains largely unknown. Moreover, it is likely no studies have been conducted on investigating the pangolins’ potential distribution range shift in responding to the Pleistocene climate fluctuations due to the extreme difficulty of collecting occurrence data on this nocturnal and elusive species. We still lack a comprehensive understanding of the old and recent evolutionary history of this species has prevented development of effective conservation strategies [18].

Here, we investigate the impacts of Pleistocene climate fluctuations on the demographic history and distribution of Chinese pangolins. To that end, we performed an integrative species distribution modelling (SDM) analysis with occurrence data from our field surveys and demographic histories inferred from the nuclear single nucleotide polymorphisms (SNPs) and site frequency spectrum (SFS) of the Chinese pangolin in Guangdong Province. We present insights into how Pleistocene climate fluctuations may have shaped the evolutionary history and population dynamics of the species in southern China.

## Results

### Population genetic structure

**Fig. 1:**
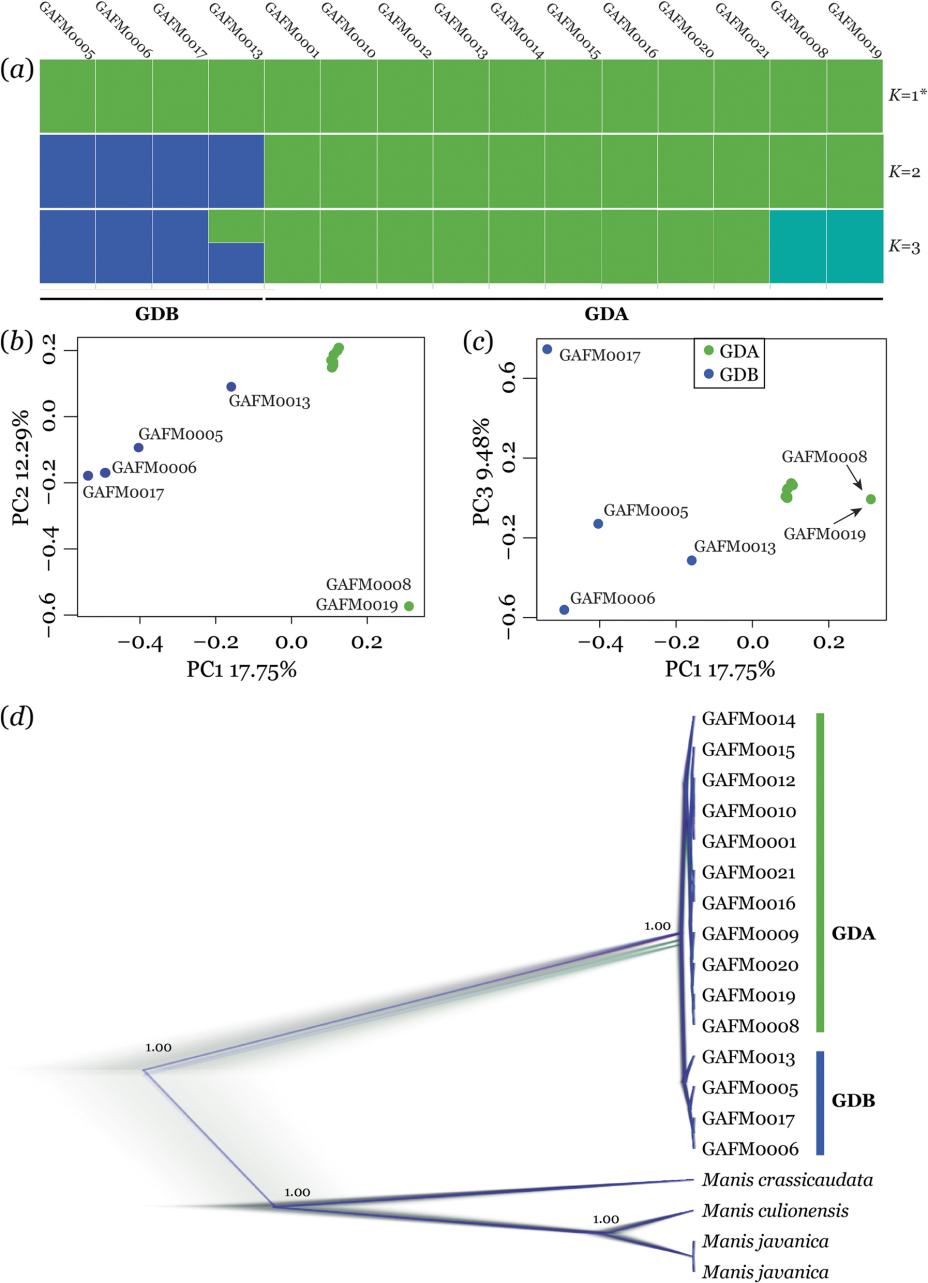
Inferred genetic structure of Chinese pangolin subpopulations of Guangdong Province according to (**a**)ADMIXTURE analysis when *K* = 2, (**b**, **c**) PCA, and (**d**) DensiTree showing 100,000 phylogenetic tree topologies inferred from whole mitochondrial genome sequences using BEAST. In (**a**), codes above and below the plot refer to individual and subpopulation, respectively. *denotes the optimal *K* value. In (**a**), (**b**) and (**c**), different subpopulations are indicated with different colours. In (**d**), blue line represents the most well-represented tree topology, followed by the red topology and finally the green. Labels on nodes indicate the posterior probabilities of the Maximum Clade Credibility Tree

The ADMIXTURE analysis showed all the individuals originated from a single population, with the lowest cross-validation error at *K* = 1 (Fig. 1 and S1). Two subpopulations of the GDA (*n* = 11) and GDB (*n* = 4) were identified when *K* = 2. The PCA plots showed the two predefined subpopulations of the GDA and GDB were clearly separated, typically in the axes of PC1 and PC3. Notably, two individuals (GAFM0008 and GAFM0019) were separated from GDA subpopulation by the axes of PC1 and PC2. We also identified the two subpopulations from the phylogenetic tree inferred based on complete mitochondrial genome sequences (Fig. 1). Additionally, the genetic differentiation index (*F*_ST_) between the two subpopulations was estimated to be 0.111. Our data provide multiple lines of evidence indicating that there were two panmictic subpopulations in this study.

### Population dynamics and divergence history

**Fig. 2:**
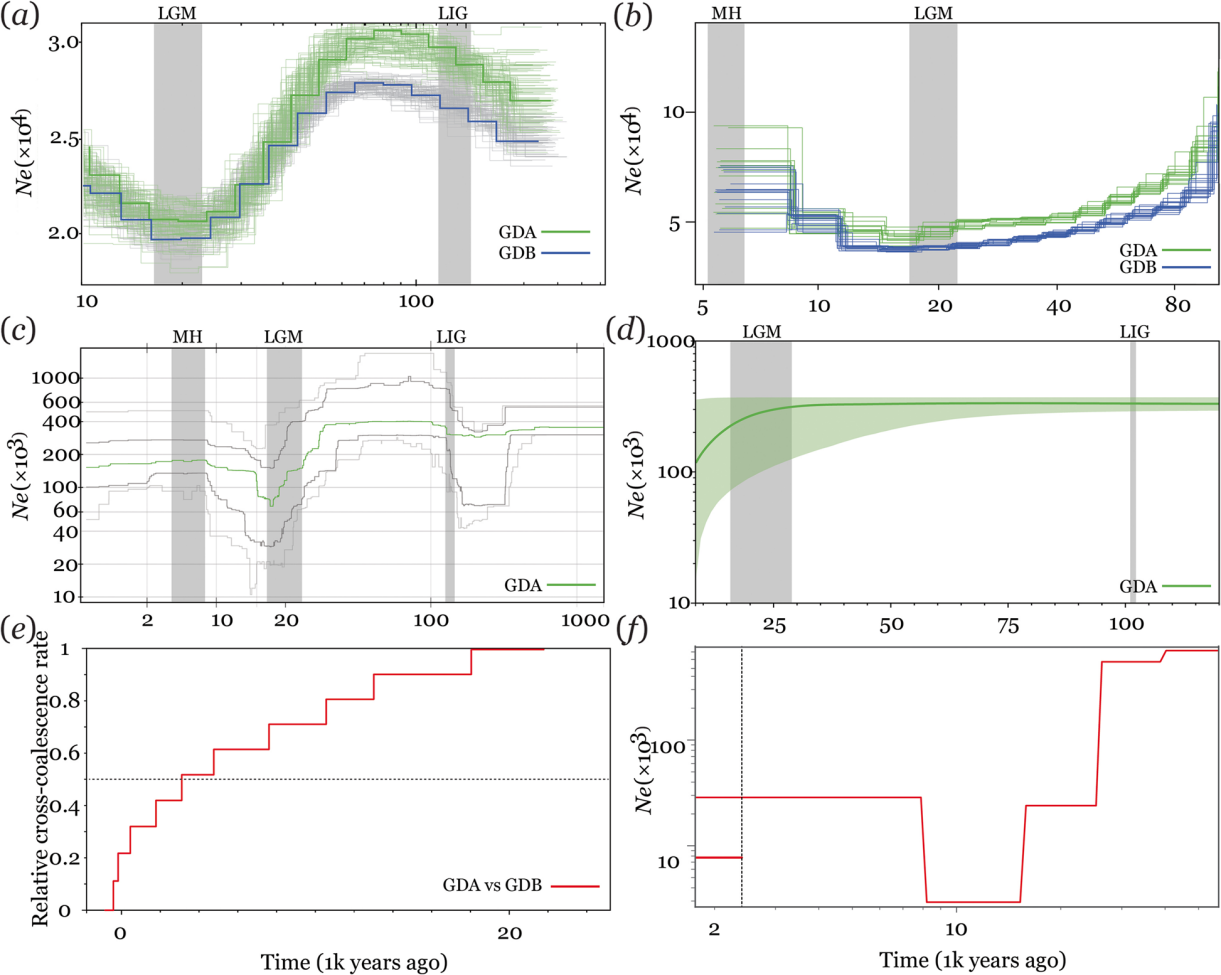
Demographic history of the studied Chinese pangolin subpopulations. **a** PSMC estimate based on the de novo assembly, and population genomic estimates from (**b**) MSMC2, (**c**) STAIRWAY PLOT and (**d**) BSP based on 10 whole mitochondrial genomes across four time periods: the current time, the Mid-Holocene (MH; 7.5–5 ka), the last glacial maximum (LGM; approximately 21 ka) and the last interglacial (LIG; approximately 140–120 ka). The thick dark green line in (**d**) indicates the average estimated trajectory, while the light green lines indicate the 90% credibility interval. (**e**) Split time inferred by MSMC2 between the GDA and GDB subpopulations. (**f**) Reconstruction of past variations in the GDA subpopulation in *Ne* and split time between GDA and GDB subpopulations inferred by SMC +  + . The detailed information in each dataset used in those analysis is described in Table S5

All the demographic analysis, including the pairwise sequentially Markovian coalescent (PSMC), the multiple sequential Markovian coalescence (MSMC2), the STAIRWAY PLOT, SMC +  + and BSP analyses showed clear signs of significant bottlenecks or strong population contractions with a marked decrease of *N*_e_ (~ 0.5 to fivefold change) during the LGM (ca. 22–15 ka). The PSMC and STAIRWAY PLOT analyses suggested population increased during the LIG (ca. 150–100, ka). Two demographic analyses (MSMC2 and STAIRWAY PLOT) revealed population stability during the MH (ca. 5.5–2 ka) (Fig. 2a and b).

The relative cross coalescence rate (RCCR) parameter implemented in MSMC2 software was used to estimate the divergence time of the two subpopulations. The results showed that the GDA and GDB subpopulations began to separate ~ 20 ka, and complete separation occurred approximately 3.0–2.5 ka (RCCR = 0.5, Fig. 2e). Furthermore, the estimated time of the separation event was highly consistent between MSMC2 and SMC +  + analysis (Fig. 2f).

### Range dynamics

**Fig. 3:**
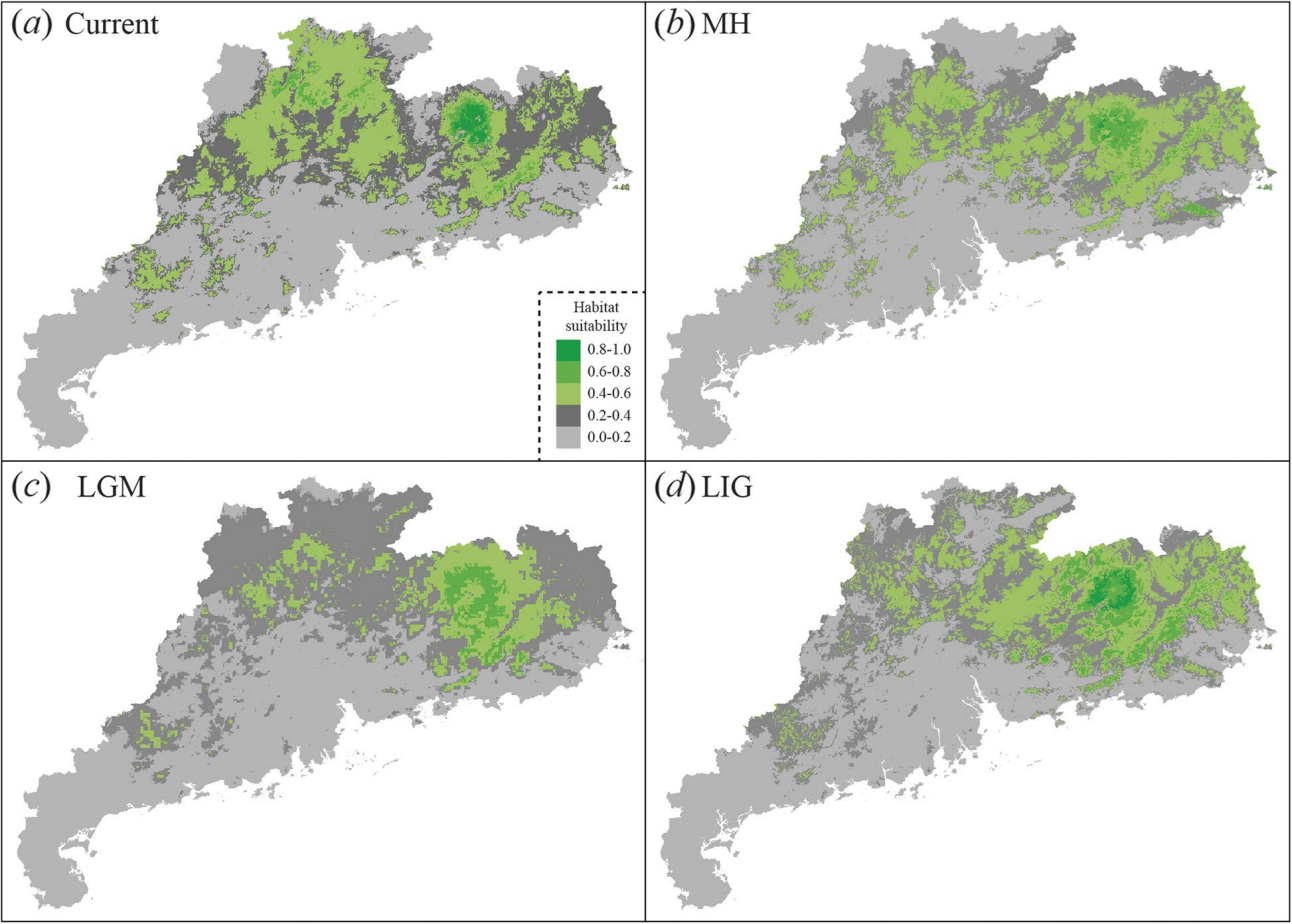
Species distribution models for the Chinese pangolin. **a** to (**d**) are the potential suitable areas in the current and historical (Mid-Holocene [MH], 5–7.5 ka, last glacial maximum [LGM], ~ 21 ka, and the last interglacial [LIG], 120–140 ka) times, respectively. Colours indicate the probability of occurrence as predicted by MAXENT [23]

**Fig. 4:**
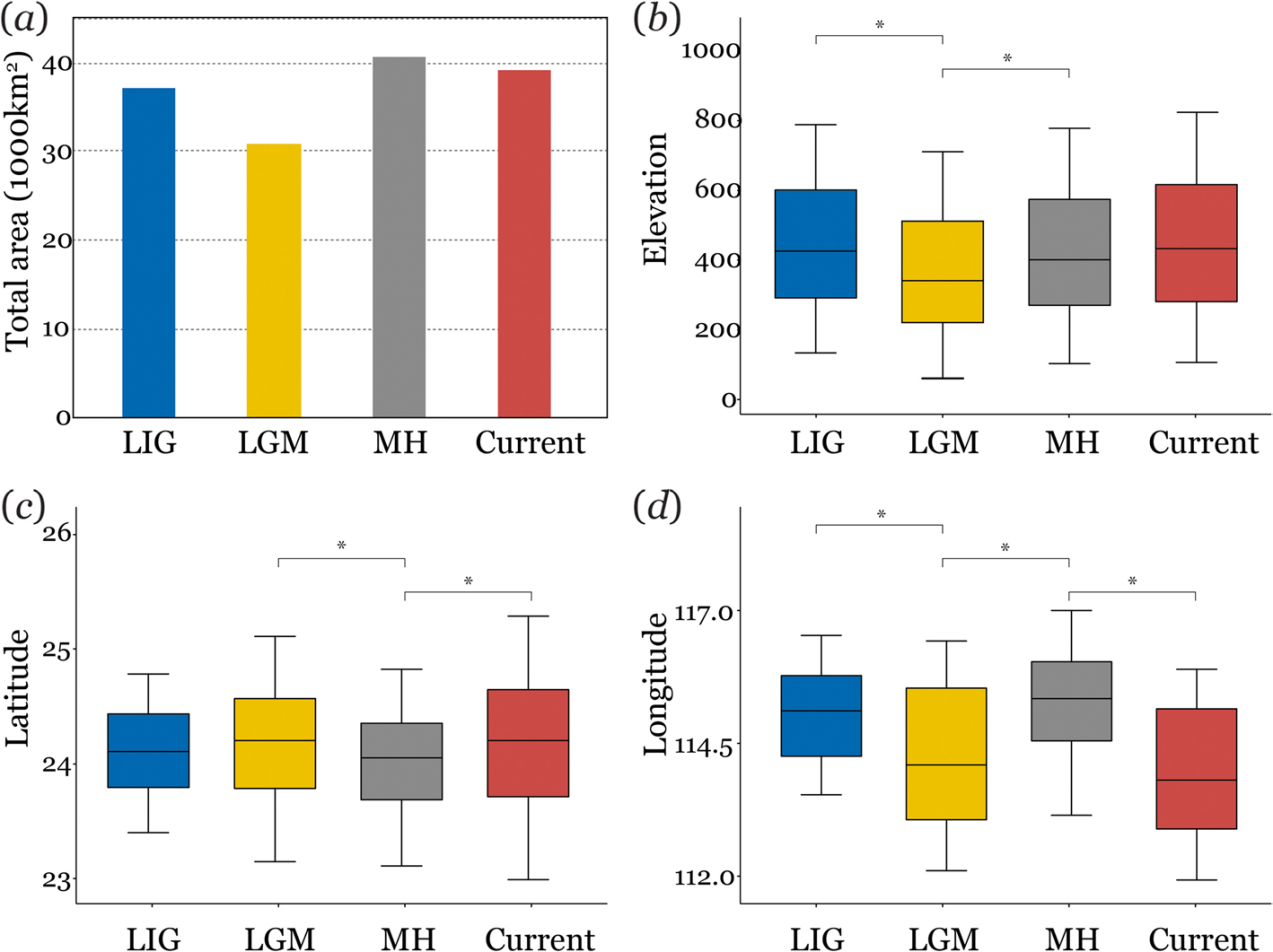
Shifts in the Chinese pangolin distribution in four time periods. **a** to (**d**) represent the total area, elevational, latitudinal, and longitudinal dimensions under climate change (over the four time periods of the LIG, LGM, MH, and Current). *Statistical differences, *p* < 0.05

Model H1 generated the lowest AIC value (Figure S2 and Table S1) and was chosen to generate the projections of the species distribution model (Fig. 3). Our distribution model accurately predicted the AUC values (mean ± SE: 0.802 ± 0.015; Table S2), indicating excellent performance of the predictive models. Overall, the suitable distribution areas shrank by 14.4% from the LIG to LGM, expanded by 31.4% from the LGM to MH, and remained stable from the MH to the present (Fig. 3). The suitable distribution areas of the northwest corner of Guangdong Province decreased significantly during the LGM. The eastern region of Guangdong Province showed the highest habitat suitability across all the climate scenarios (Fig. 3). In addition, we found the suitable habitat significant retreated to lower elevations during the LGM (Fig. 4). We detected the significant increase of suitable areas in the western side of the province from the LIG compared to the current range extent (ANOVA: *P* < 0.01, Fig. 4).

## Discussion

In our study, the fifteen individuals from Guangdong province were assigned into a single population by the ADMIXTURE analysis. However, two subpopulations (GDA and GDB) were revealed by the results of genetic distances, phylogenetic tree and principal component analysis. However, we could not accurately assign these two subpopulations to their geographic distributions due to the lack of accurate sampling site information for most samples. Given the descriptions of eight individuals’ sampling localities, we expect GDA to have originated in northern regions of Guangdong Province and GDB to have originated in the south (Table S3). The total suitable areas kept stable from the MH to present inferred from SDM analysis, but the two subpopulations began to separate at ~ 20 ka and diverged in recent years following the MH. Thus, we argue that the separation of the two subpopulations might have been initiated by cold temperatures in the LGM, and the complete split was probably facilitated by human stressors [18, 24]. Our findings here are based on occurrence data to infer past climate, but it should be noted that other nonhuman biotic factors (e.g., biological invasion [25] and diseases [26]) could have played a role in inferred population contractions, but we could not evaluate this due to lack of evidence.

Pleistocene climate fluctuations strongly influenced demographic processes and the distribution of species in Europe and North America [2, 3]. However, the effects of Pleistocene climate fluctuations on demographic processes and the distribution of the species in southern China have remained controversial. Some studies have indicated that the climatic conditions of the LGM period have shaped the genetic diversity of various species (e.g., [16, 17]), whereas other studies have produced contradictory evidence (e.g., [14, 15]). Two possible reasons for these opposing results could be methodological: the estimation of demographic history based on limited genetic data may lead to biased inferences [27, 28], and interpreting either historical population size change or SDMs independently is problematic and potentially misleading [29]. Here, we infer the history of the Chinese pangolin by reconstructing temporal changes in effective population size using multilocus datasets and to elucidate historical causes of the distribution pattern of Chinese pangolins based on large amounts of occurrence data from field surveys. Our results revealed that the relatively warmer climate conditions in the LIG triggered the increase in Chinese pangolin population size and potential habitat expansion, whereas the dramatic cooling and drying climate of the LGM caused a severe population decline, potential habitat contraction, and an elevational gradient shift. A similar pattern has been observed in other organisms in East Asia [18], and in the two Chinese pangolin populations from Yunan Province, in which population size decline was revealed from approximately 120–10 ka and 25–13 ka, respectively [22]. Ice sheets did not cover most areas in southern China, the temperatures were 2–4 °C colder than today during the LGM. Furthermore, the Chinese pangolin is considered to be an extremely temperature-sensitive species, and sharp decrease in temperature may cause pneumonia leading to death in this species [18]. Thus, these patterns could be explained if the cold temperatures reduced the adaptive capacity of this species, leading to a decline in population size and genetic diversity during the LGM. This is in accordance with the finding that the mean annual temperature (BIO1) climatic factor showed the top contribution rate among all variables (Table S4).

The Chinese pangolin has been widely distributed in southern China over the last century, typically inhabiting forests with dense shrubs in warm subtropical zones [18–20]. Due to overexploitation from heavy poaching and trafficking, the status of the Chinese pangolin is critically endangered with a declining trend [19]. Fortunately, we obtained a number of photo or video records of wild Chinese pangolins by using infrared cameras in more than ten counties and/or nature reserves in Guangdong Province since 2019. The predicted potential suitable habitat area at present was observed to be generally similar to the actual known distribution area in Guangdong Province. Furthermore, the suitable habitats are generally large and connected, accounting for 22% (39,202/177,900 km^2^) of the total area of Guangdong Province. This suggests there is potential for population recovery of Chinese pangolin in Guangdong Province. In addition, our study could provide helpful guidance for detecting key habitats of populations in Guangdong Province. For instance, we elucidated a stable habitat region in eastern Guangdong Province and revealed a wide range of suitable habitats across climate change scenarios. The population size increased when the climate was warmer than the present day during the MH. We speculate that climate warming in the future should shift Chinese pangolin range towards high altitudes, but it may encounter more fragmented habitats there. In addition, when combining the continued poaching pressure, their population is likely to suffer further under the global climate warming. Therefore, we suggest that priority should be given to the conservation and restoration of the Chinese pangolin's current habitat and the implementation of effective anti-poaching measures to better improve population recovery, especially in areas of highly suitable habitat such as forests of the eastern region of Guangdong Province. In addition, more samples from the whole distribution range of this species will be added to detect the status of the genetic structure and the mechanism of endangerment in further research.

## Conclusion

Our results revealed that population size, suitable areas and range elevation of the Chinese pangolin decreased during the LGM period, suggesting that Pleistocene climate fluctuations played an important role in shaping the genetic diversity and range distribution within this species. The human stressors that contributed to the recent divergence of two Chinese pangolin subpopulations in Guangdong Province may have been initiated by Pleistocene climate change ~ 20 ka. We also identified a wide range of suitable habitats across climate change scenarios in eastern Guangdong Province, and a key protection area for this species could thus be established. As such, this study provides a more thorough understanding of the impacts of Pleistocene climate fluctuations on a mammalian species in southern China and suggests more robust management and conservation plans for this critically endangered species of special interest.

## Methods

### Occurrence record collection

**Fig. 5:**
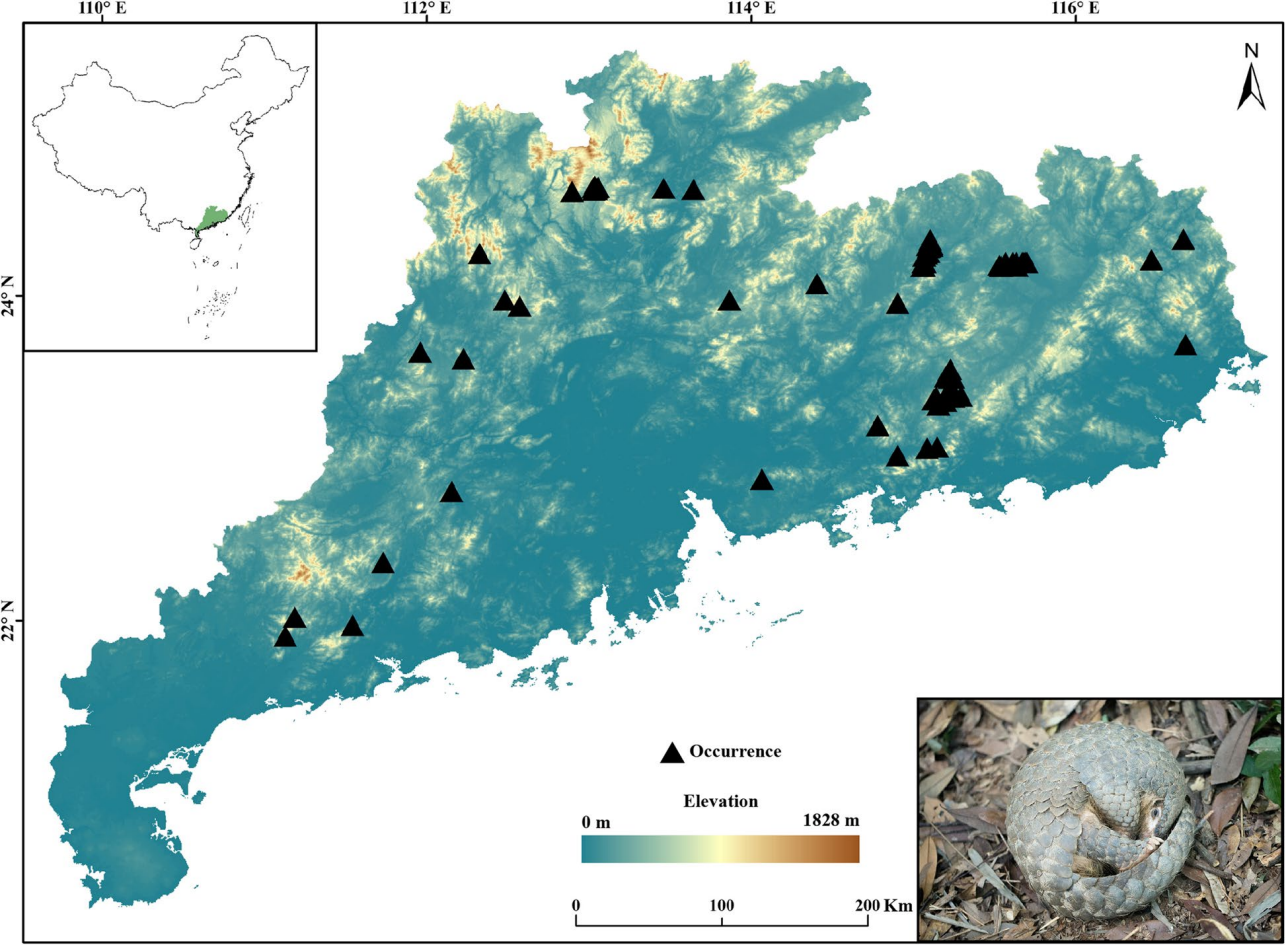
Map showing the occurrence points of Chinese pangolins in Guangdong Province, China

Guangdong Province in southern China, which is the main distribution area of Chinese pangolins [17, 18]. We obtained a total of 470 occurrence records of Chinese pangolins in Guangdong Province from our field surveys by recording the location of their burrows. First, we discarded records with obvious georeferencing errors (i.e., located in water bodies), as well as duplicate records. To avoid overfitting in the model, when multiple records occurred at a spatial resolution of 1 × 1 km, we retained only one record using the “spatially rarify occurrence data for sdms” function implemented in the SDM toolbox [30]. The final dataset contained 73 occurrence data points which all came from our field surveys (Fig. 5).

### Whole-genome resequencing data processing and SNP calling

We downloaded the whole-genome resequencing reads of 15 Chinese pangolin individuals from Guangdong Province (China National GeneBank DataBase: accession number CNP0001723) for analysis in this study. We used BWA v0.7.17 [31] to align the trimmed paired-end reads of 15 individuals against the Chinese pangolin reference genome [22]. The sequencing depth ranged from 13.17 to 22.85, with an average depth of 17.03 ± 2.43. We generated binary sequence alignment files using SAMTOOLS v.1.7 [32] and removed PCR duplicates with PICARD tools (http://picard.sourceforge.net). We performed the InDel realignment with the IndelRealigner algorithm implemented in the Genome Analysis Toolkit (GATK) v.3.5.0 [33]. We called variants using both the mpileup command in SAMTOOLS v.1.7 and GATK UnifiedGenotyper with the default settings. We selected concordant common sites using the SelectVariants package in GATK with the default settings. We performed hard filtering of the obtained variants with the following parameters: “QUAL < 30.0 || QD < 2.0 || FS > 60.0 || MQ < 40.0 || MQRankSum < -12.5 || ReadPosRankSum < -8.0” [34], because it produces more high-quality SNPs than the default settings. A total of 5,402,440 biallelic SNPs were retained. For population genetic analyses, we also filtered reads with a depth of less than 10X and filtered SNPs with a depth distribution of all sites less than 2.5% or more than 97.5%. We excluded the SNPs with a minor frequency allele (MAF) < 0.05 and more than 20% missing data across all individuals. Finally, we filtered out the SNPs with excess heterozygosity and that showed highly significant deviation from Hardy–Weinberg Equilibrium (*P* < 0.001) by the filter expression "ExcHet < 0.05 and HWE < 0.001, respectively" using BCFtools [32]. The final retained dataset included 1,557,701 informative SNPs for ADMIXTURE, *F*_ST_, MSMC2, SMC +  + analyses and PCA. For the STAIRWAY PLOT analyses, the SNPs were not filtered for MAF. However, missing data was not allowed to avoid distorting the allele frequency spectra. More details on each dataset are given in Table S5.

### Population genetic structure

To examine the patterns of genetic structuring among Chinese pangolin populations, we used four different approaches, ADMIXTURE, PCA, *F*_ST_ and mitochondrial phylogeny tree construction. Population structure was analysed using ADMIXTURE software [35]. The number of populations (*K*) was predefined from 1 to 5, and the optimal clustering number was determined according to the minimum value of the cross-validation error rate. Principal component analysis (PCA) was performed using GCTA software [36] with the default parameters and settings. The *F*_ST_ estimation was calculated using Weir and Cockerham’s fixation index [37] with a nonoverlapping 50-kb window in VCFtools [38]. Furthermore, we obtained the complete mitochondrial genome from the published sequencing data of 15 Chinese pangolin individuals, by filtering and assembling clean paired-end reads using SPADES v.3.11.0 software [39] with the Chinese pangolin (GenBank accession number: MT335859) as a reference. We estimated the phylogenetic relationships using the complete mitochondrial genome by Bayesian inference (BI), with *M. javanica* (KP_306515, NC_026781), *M. culionensis* (NC_036434), and *M. crassicaudata* (NC_036433) as outgroups, which was performed with BEAST v.2.5 [40]. The best substitution model, GTR + G, was selected using MODELTEST v.3.7 [41]. We conducted two independent runs of Markov Chain Monte Carlo (MCMC) analyses for 100,000,000 generations with sampling every 1,000 generations. We visualized the Maximum Clade Credibility trees and posterior probabilities at nodes applied to the DENSITREE v.2.2.6 [40] after discarding the first 10% of each MCMC chain as burn-in.

### Population demographic and divergence history

To infer the detailed historical changes in effective population size (*N*_e_) and the divergence time between the two genetic Chinese pangolin subpopulations (subpopulation GDA [*n* = 11] and subpopulation GDB [*n* = 4]) as inferred by the population structure analysis (see Results). We employed a combined strategy involving five complementary algorithms. We first used the individual genome-based pairwise sequentially Markovian coalescent (PSMC) to reconstruct historical changes in the effective population size [42]. For the PSMC analysis, which utilized LD information, we selected individuals with the highest sequencing depths from the two subpopulations (GAFM0020 for GDA, GAFM0005 for GDB). To determine the variance in *Ne* estimates, the PSMC plot and an additional 100 bootstraps replicates were generated with the following set of parameters: -N30 -t 15 -r 5 -p 4 + 25*2 + 4 + 6. For MSMC2 analysis [43], we first estimated population size fluctuation over time. We generated aligned reads (in BAM format) from sample-specific VCF and mask files using *bamCaller.py* from msmc-tools (available at https://github.com/stschiff/msmc-tools). We prepared mappability masks for the reference genome following the procedure of Heng Li’s SNPable program (http://lh3lh3.users.sourceforge.net/snpable.shtml). We phased the variants with SHAPEIT [44], generated the input using *multihetsep.py* and generated 20 bootstrap runs using *multihetsep_bootstrap.py* from msmc-tools. To infer the divergence times of the two genetic subpopulations by MSMC2, we first selected two individuals with less than 10% of missing call rate from each of the two genetic subpopulations for calculation using MSMC2 [43]. Then, we set the calculation parameters as follows: –skipAmbiguous -I 0–4, 0–5, 0–6, 0–7, 1–4, 1–5, 1–6, 1–7, 2–4, 2–5, 2–6, 2–7, 3–4, 3–5, 3–6, 3–7 -i 20 -t 6 -p ‘10*1 + 15*2’. The results were visualized with R scripts (available at https://github.com/stschiff/msmc-tools). For the STAIRWAY PLOT analysis [45], we estimated the folded site frequency spectrum (SFS) data from the 5,402,440 biallelic SNPs with the same filtering settings for the population genetic analyses except only kept no missing data SNPs using ANGSD v.0.928 [46]. The final dataset for STAIRWAY PLOT analysis for the GDA subpopulation retained 1,001,672 SNPs. The STAIRWAY PLOT and its variance were estimated from 200 bootstrap SFSs with the default 2/3 of the data used for training and (5, 10, 15 and 20) as the number of random breakpoints. We did not perform STAIRWAY PLOT, SMC +  + and BSP analyses of GDB (*n* = 4) because the limited sample size might result in a risk of producing a false-positive due to the structure effect [47]. Furthermore, we used SMC +  + to estimate the population size trajectories and divergence time of the GDA and GDB subpopulations, because it does not depend on phase data, and the calculation bias introduced by switch errors during phasing analysis can be avoided. We used the *vcf2smc* subcommand to convert SNP data to SMC +  + format. Then, we used the *estimate* subcommand to calculate the effect population size as a function of time with the default parameters. We performed 20 independent replicate analyses to obtain more reliable results. Next, we created an SMC +  + pairwise dataset using the *vcf2smc* subcommand. We estimated the divergence time using the command *split* with the default parameters. For all analyses, we set the mutation rate (*μ*) per generation as 1.47 × 10^–8^ [24] and the generation time as one year [48].

Furthermore, species show quantitative variation between mitochondrial and nuclear genomes that can lead to highly discordant evolutionary histories [49]. Here, we generated a Bayesian skyline plot (BSP) which reconstructs changes in the effective population size based on the complete mitochondrial genome across evolutionary time in BEAST v.2.4.5. The substitution rate (estimated in the divergence time analysis) was set by a normally distributed prior with a mean of 0.033 substitutions/site/million years and a standard deviation of 0.01 substitutions/site/million years [50]. We tested the suitable substitution models for each population in MODELTEST v.3.7. Settings similar to those used for constructing the phylogenetic tree were used, as the exception that the prior tree was set as the coalescent: Bayesian skyline. Validation of the successful model convergence under the listed parameters was assessed in TRACER v.1.7 [23] to ensure that the effective sample size (ESS) value was greater than 200.

### Species distribution modelling (SDM)

To investigate the possible influence of climatic changes on the distribution of Chinese pangolins. We downloaded 19 bioclimatic layers from four-time periods: the current time, the Mid-Holocene (MH; 7.5–5 ka), the last glacial maximum (LGM; approximately 21 ka), and the last interglacial (LIG; approximately 140–120 ka) as environmental predictors at a 30-arcsec (~ 1 km) resolution from the WorldClim database (http://www.worldclim.org/ ; [51]). We used three GCMs (CCSM4, MIROC-ESM, and MPI-ESM-P) for both the Mid-Holocene and LGM climate scenarios, but only one GCM (CCSM) was available for the LIG. We selected BIO (bioclimatic variables) parameters using PCA to avoid multicollinearity. The results showed that the variance in climate of the study area could be explained by five principal components (PCs) that captured 90% of the variance in the data (Table S6). Thus, we selected one representative BIO parameter per PC (BIO1 = annual mean temperature, BIO6 = minimum temperature of coldest month, BIO8 = mean temperature of wettest quarter, BIO17 = precipitation of driest quarter, and BIO19 = precipitation of coldest quarter, Table S1) to generate the SDMs. We generated SDMs using the maximum entropy algorithm in Maxent v.3.3.3e [52]. To set the proper Maxent options and settings, we searched for the appropriate combinations of feature classes (determining the shape of the response curves) and regularization multipliers (determining the penalty for adding parameters in the model) by evaluating model scores based on the Akaike information criterion (AICc). We identified the best model using the ENMeval package [53] with the “ENMevaluate” function in R v.3.3.3 [54]. The model feature types used were “L”, “H”, “LQ”, “LQH”, “LQHP”, “LQHTP” (where: L = linear, Q = quadratic, H = hinge, P = product and T = threshold) and the regularization (RM) values (0, 0.5, 1, 1.5, 2, 2.5, 3, 3.5, 4). To identify a proper evaluation, we randomly selected 75% of the localities to generate a training set, and reserved the remaining 25% to test the validity of the models. We set the convergence threshold to 10^–5^ and the maximum number of iterations to 500 [55]. We used ArcGIS Desktop version 10.3 (ESRI) visualize the spatial environmental data and model output.

### Quantifying climate change impacts

To quantify the influence of Pleistocene climate fluctuations on the potential distribution of the Chinese pangolin, we ran 20 cross-validation replications of each model and obtained ensemble distribution predictions by weighing them with their areas under the receiver operating characteristic curves (AUCs). The AUC values varied from 0.5 (complete randomness) to 1 (perfect discrimination), where 0.7–0.8 indicated acceptable performances, 0.8–0.9 indicated excellent performances, and > 0.9 indicated outstanding performance [56]. Next, we averaged the outputs for the MH and LGM to obtain a suitability map for all projections. We extracted the suitability values from the modelling results and used the maximum training sensitivity plus specificity threshold (MaxSS) to classify the average continuous probabilities into binary maps for each timeframe. Then, we calculated the total area of suitable habitats under all projections. In addition, we extracted the elevations, latitudes, and longitudes of the “present” grid cells, and one-way ANOVAs were conducted to identify their variations among the projections for the four periods in R.

## Supplementary Information

**Additional file 1:** **Table S1.** Best fit MAXENT model based on delta AICc from ENMeval. **Table S2.** AUC values for each model. **Table S3.** Sampling site data, gender, distribution clustering, and missing data rate of genetic samples of Chinese pangolins used in this study. **Table S4.** Estimation of the relative contributions of the environmental variables to the Maxent model. **Table S5.** Summary of each dataset used for the respective analysis. Table S6. Bioclimatic variable selection for species distribution modelling based on PCA.

**Additional file 2:** **Figure S1.** Cross validation (CV) error plot for ADMIXTURE analysis. **Figure S2.** Delta AICc values of all models compared in ENMeval. Model H 1 was the best fit model.

## Data Availability

The whole-genome resequencing reads used and/or analysed during the current study are available in the China National GeneBank DataBase (accession number CNP0001723). The VCF files and SFS data for population genetic structure and demographic history analysis, and the mitochondrial genome sequences are available in the Science Data Bank (https://www.scidb.cn/cstr/31253.11.sciencedb.01679). Locality information is not publicly available due to the risk of pangolin poaching and trafficking but is available from the corresponding author on reasonable request.

## Abbreviations

LIG: Last interglacial
LGM: Last glacial maximum
MH: Mid-Holocene
SDM: Species distribution modelling
BIO: Bioclimatic variables
PCs: Principal components
BSP: Bayesian skyline plot
PSMC: Pairwise sequentially Markovian Coalescent
MCMC: Markov Chain Monte Carlo
MSMC2: Multiple sequentially Markovian Coalescent 2
*N*_e_: Effective population size
AICc: Akaike information criterion
AUC: Area under the receiver operating characteristic curve
PCR: Polymerase chain reaction
ka: Thousand years ago
*μ*: Mutation rate
