# Supplementary material for: Influence of Pleistocene climate fluctuations on the demographic history and distribution of the critically endangered Chinese pangolin (Manis pentadactyla)
Source: BMC Zool. 2022 Sep 1;7:50. doi: 10.1186/s40850-022-00153-6 (PMC10127079; doi:10.1186/s40850-022-00153-6)
Supplement: Supplementary file 2 — Additional file 2: Figure S1. Cross validation (CV) error plot for ADMIXTURE analysis. Figure S2. Delta AICc values of all models compared in ENMeval. Model H 1 was the best fit model. [file 40850_2022_153_MOESM2_ESM.docx]

**Supplementary Materials**


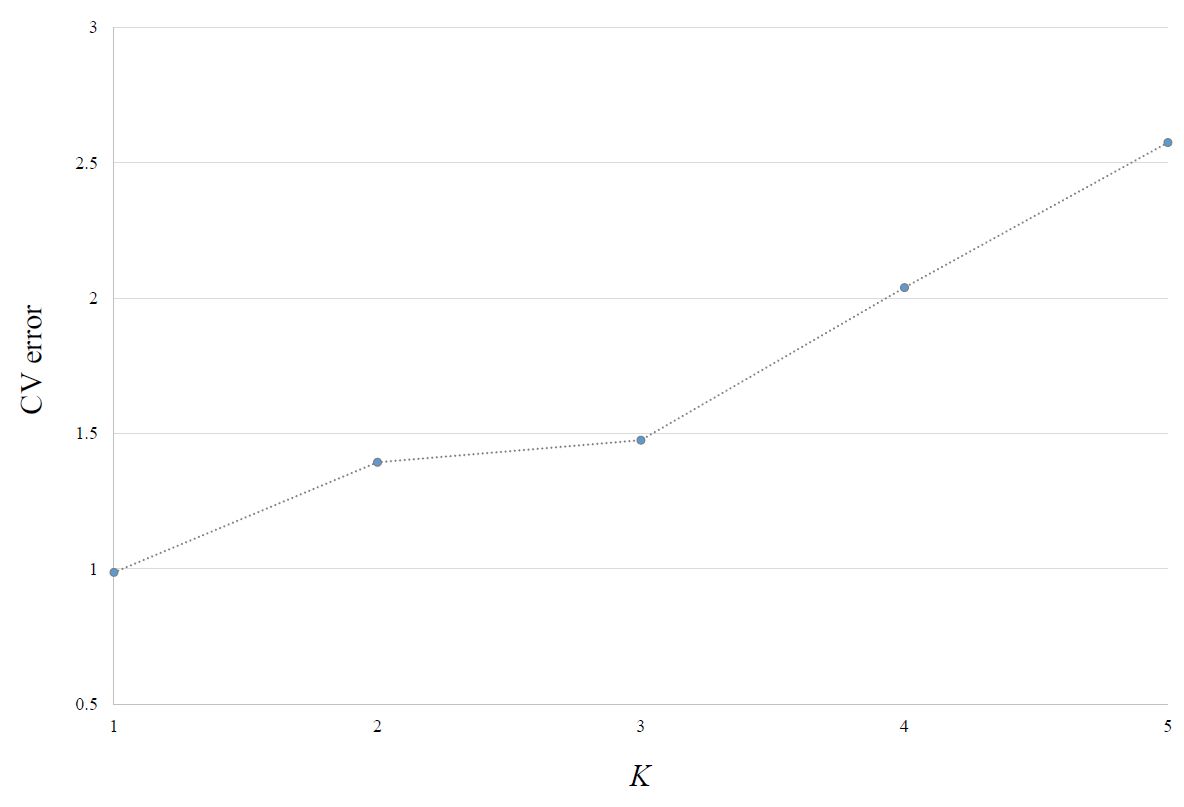


**Figure S1**. Cross validation (CV) error plot for ADMIXTURE analysis. Optimal *K* = 1.


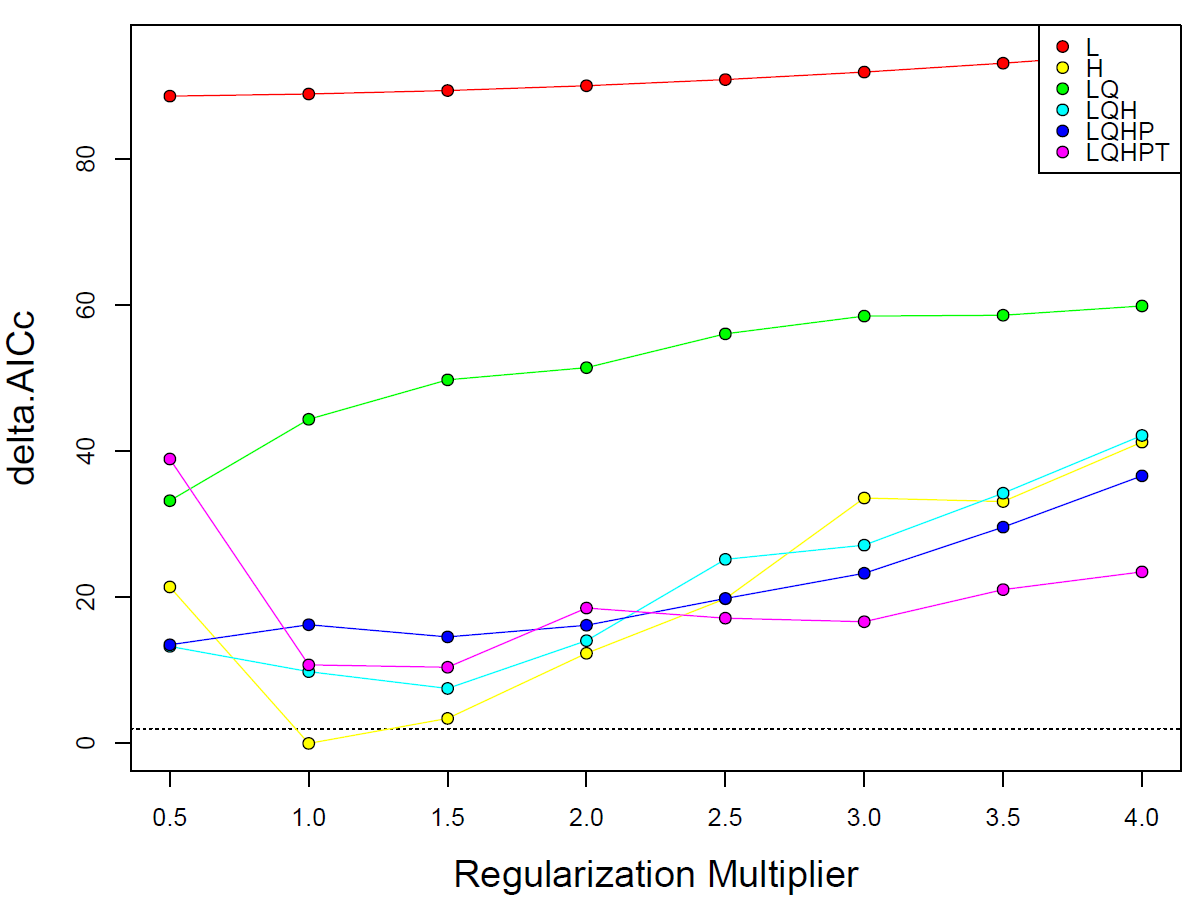


**Figure S2**. Delta AICc values of all models compared in ENMeval. Model H 1 was the best fit model.
